# Elevated serum interleukin-6 is predictive of coronary artery disease in intermediate risk overweight patients referred for coronary angiography

**DOI:** 10.1186/s13098-017-0266-5

**Published:** 2017-09-06

**Authors:** Marco V. Wainstein, Márcio Mossmann, Gustavo N. Araujo, Sandro C. Gonçalves, Gabriela L. Gravina, Marlei Sangalli, Francine Veadrigo, Roselene Matte, Rejane Reich, Fernanda G. Costa, Michael Andrades, Antônio Marcos V. da Silva, Marcello C. Bertoluci

**Affiliations:** 1 Programa de Pós-Graduação em Medicina: Cardiologia, Universidade Federal do Rio Grande do Sul, Porto Alegre, Brazil; 2 Serviço de Cardiologia do, Hospital de Clínicas de Porto Alegre, Porto Alegre, Brazil; 3 Serviço de Medicina Interna do, Hospital de Clínicas de Porto Alegre, Ramiro Barcelos 2350, Porto Alegre, RS 90035-003 Brazil; 4 Faculdade de Medicina da, Universidade Federal do Rio Grande do Sul, Porto Alegre, Brazil; 5 Unidade de Análises Moleculares e de Proteínas (UAMP), Hospital de Clinicas de Porto Alegre, Porto Alegre, Brazil; 6 Departamento de Fisioterapia e Reabilitação, Universidade Federal de Santa Maria, Santa Maria, Brazil; 7 Programa de Pós-Graduação em Medicina Ciências Médicas, Universidade Federal do Rio Grande do Sul, Porto Alegre, Brazil

**Keywords:** Interleukin-6, Risk factors, Risk scores, Coronary angiography, Inflammation, Coronary artery disease

## Abstract

**Background:**

Interleukin-6 (IL-6) plays a central role in atherosclerosis and inflammation. It may improve risk prediction in patients at intermediate cardiovascular risk.

**Objective:**

To analyze the impact of serum IL-6 in predicting early angiographic coronary artery disease in patients at intermediate cardiovascular risk with chest pain.

**Methods:**

In a cross-sectional study, patients referred for coronary angiography due to suspected coronary artery disease (CAD) were included. Coronary artery disease was defined as the presence of at least 30% stenosis in one or more coronary artery. Severity of CAD was classified by the anatomic burden score. Performance of serum IL-6 assay was compared with ACC/AHA atherosclerotic cardiovascular disease (ASCVD) risk score and hs-CRP through receiver operating characteristic (ROC) curves.

**Results:**

We have included 48 patients with a mean 10-year ASCVD risk of 10.0 ± 6.8%. The prevalence of CAD was 72.9%. The presence of CAD was associated with higher mean levels of IL-6 (p = 0.025). Patients with CAD had significantly more overweight than subjects without CAD. In 27% of patients, IL-6 was >1.0 pg/mL and 100% of these patients had CAD, while only 64% in those with IL-6 <1.0 pg/mL, corresponding to a positive predictive value of 100% (p = 0.015). The area under the receiver operating characteristic (ROC) curve of IL-6, hs-CRP and ASCVD were respectively 0.72, 0.60 and 0.54. Intermediate risk patients with IL-6 >1.0 pg/mL were further reclassified into ASCVD high risk due to the presence of coronary lesions.

**Conclusion:**

In intermediate risk patients referred for coronary angiography, a serum IL-6 level above 1 pg/mL is predictive of significant CAD. IL-6 determination may be useful to reclassify ASCVD intermediate risk patients into higher risk categories.

## Background

Coronary artery disease (CAD) is the main cause of death globally, with an increasing prevalence in the western world [1, 2]. The precision in estimating the risk for future CAD is crucial for treatment decision and prevention strategies. Improving accuracy in risk prediction is especially important among patients at intermediate cardiovascular risk, where calibration is limited when using global risk score calculators [3]. The 2013 ACC/AHA guidelines currently suggest the use of 10 year ASCVD risk score to estimate the risk for future coronary events and to define strategies of primary prevention [4]. Although this approach is important, the precision in patients at intermediate coronary risk is less accurate than in higher or lower risk categories [5].

The prevalence of coronary disease in patients who are referred for coronary artery angiography due to chest pain is usually high [6]. However, chest pain can be managed with distinct approaches according to specific assistance protocols in different health care systems. In general, middle-aged non-diabetic individuals who have few cardiovascular risk factors are considered at intermediate-high risk, and the presence of chest pain in these patients poses an important challenge for the health care team. The incidence of false positives for coronary disease tends to be higher in this category than in patients at high-risk. Therefore, the use of emergent biomarkers may help in risk reclassification, being an interesting approach.

There is extensive evidence supporting a role of inflammatory response in the pathophysiology of acute coronary syndromes (ACS) and in the natural history of atherosclerosis [7–9]. Cardiovascular events are more common in patients with high circulating levels of several inflammatory markers, and treating based on inflammatory parameters, such as hs-C reactive protein (hs-CRP), have been proved to reduce outcomes [10]. Interleukin-6 (IL-6), however, is a central mediator of the acute-phase response and a primary determinant of hepatic production of C-reactive protein (CRP) [11]. IL-6 is associated with increased incidence of myocardial infarction and mortality among patients with ACS [12, 13]. As hs-CRP has been classically linked to coronary events [14], it is also reasonable to address the role of IL-6, an even more precocious biomarker of inflammation than hs-CRP [15], in improving the detection of CAD and the severity of disease in this population.

The present study aimed to analyze the predictive value of IL-6 for diagnosing the presence of early CAD through coronary angiography in intermediate risk patients with chest pain, comparing its performance to hs-CRP. We hypothesized that IL-6 could improve the accuracy of traditional risk scores such as ASCVD and hs-CRP in patients at intermediate risk.

## Methods

### Study design and patients

We have conducted a prospective cross-sectional study with patients referred for coronary angiography due to non-acute chest pain in a reference cardiology center at Hospital de Clínicas of Porto Alegre, Brazil. Between 2013 and 2014 all patients referred to elective coronary angiography for non-acute chest pain were screened. Patients who fulfilled inclusion and exclusion criteria and accepted to participate were included in the study. All participants were in agreement to have their data published and signed a written informed consent, revised by the local ethics committee which than approved the study protocol.

Inclusion criteria were: age between 40 and 70 years and chronic chest pain. We have excluded patients with a known history of diabetes, previous acute coronary syndrome or stroke, previous coronary artery revascularization, estimated glomerular filtration rate below 45 mL/min/L. 73 m^2^, presence of class IV-NYHA congestive heart failure, chronic obstructive pulmonary disease, body mass index above 44 kg/m^2^, previous organ transplantation, current evaluation for any organ transplantation and those with presence of rheumatic, endocrine or infectious chronic diseases. We also excluded patients using any medication that could modify glucose-insulin metabolism such as insulin, metformin, sulfonylureas and patients using corticosteroids, HIV anti-retrovirals, carbamazepine, phenytoin, drugs for cancer, immunosuppressor drugs, nitrofurantoin, anti-malarics, lithium and anti-psicotic drugs.

### Clinical and biochemical investigation

Blood pressure was measured at the catheterization laboratory after 15 min of rest, in the sitting position, in the right arm, in 3 sequential measurements, using an automatic aneroid sphygmomanometer (OMRON Comfort III Visomat Incoterm, Germany). We considered the lowest blood pressure result as the final measure. Blood samples were collected between 12 and 24 h after coronary angiography. For serum IL-6 measurement, a custom Luminex^®^ assay was employed (Invitrogen^®^, #LHB0001CM) following the manufacturer orientation. Briefly, 50 µL of undiluted sample was added in a well containing buffers and magnetic beads. After 2 h of incubation (550 rpm, room temperature), the wells were washed and the detection antibody was added for further 1 h. After washing, streptavidin-RPE was added for further 30 min, the wells were washed again and the beads were suspended in 125 µL of wash buffer. Beads were read in Luminex^®^ x-Map 200 and a minimum of 100 events were recorded for each bead. The limit of detection was defined by the lowest standard value (0.08, pg/mL). Values were expressed as pg/mL.

Serum high sensitive C-reactive protein (hs-CRP) measurements were determined through turbidimetric immunoassay method (Roche^®^). Serum creatinine (Jaffé method), lipid profile, glycated hemoglobin (HPLC) were also measured.

### ASCVD risk score

The atherosclerotic cardiovascular disease (ASCVD) risk score was calculated based on AHA/ACC 2013 guidelines to estimate the 10-year risk score for men and women from 40 to 79 years of age for a first hard ASCVD event. The variables to estimate each patient risk included were age, gender, race, total cholesterol, high-density lipoprotein cholesterol, systolic blood pressure, actual treatment for high blood pressure, diabetes and current smoking status [3]. The ASCVD risk score calculator was obtained at: http://tools.acc.org/ASCVD-Risk-Estimator/. We considered intermediate risk when the 10 years score was between 7.5 and 20%.

### Coronary artery angiography parameters

Coronary angiography was performed using the Axiom Artis Siemens^®^ equipment (Germany) in all patients. Two experienced interventional cardiologists who were blinded to all other clinical variables made all angiographic measurements. Angiographic analyses were made by visual (non-quantitative) estimates of luminal narrowing in at least two different orthogonal projections.

The presence of CAD was defined as any lesion causing more than 30% reduction in  artery diameter of any epicardial vessel. We considered not significant coronary disease (NCAD) when lesions were undetectable or below to 30% stenosis.

The severity of CAD was further assessed through the “anatomic burden score” obtained from the COURAGE trial [16]. This score consisted in a grading scale of 17 progressive degrees of severity starting from zero, corresponding to complete absence of coronary disease with stenosis above 50%, to 17, which corresponds to severe 3-vessels disease, including lesions at proximal left anterior descending artery, plus left circumflex artery and right coronary involvement. To meet criteria, each lesion must represent at least 50% diameter stenosis. We divided patients into three groups: NONE for patients with score zero; MILD–MODERATE, for patients with score 1–5, corresponding to isolate lesions in right coronary artery, left circumflex artery and left anterior descending artery, increasing score in this order; and SEVERE, for patients with scores between 6 and 17, corresponding to two-vessel and three-vessel artery disease.

### Statistical analysis

Continuous variables with parametric distribution were expressed as mean ± standard deviation, whereas non-parametric variables levels were expressed as median [95% confidence interval (CI)] and analyzed using Mann–Whitney’s test. Categorical data were expressed as frequencies and their differences were analyzed using the Chi-squared test in the general characteristic Table 1. Receiver operating characteristic (ROC) curves were used to evaluate the discriminatory power of IL-6 to determine CAD. The severity of CAD (burden score) analysis was performed through ANOVA with Bonferroni post-tests. Comparison of ROC curves was performed by comparing area under the curves through trapezoid method and C statistics. We used the following cut-off values for analysis: IL-6: 1.0 pg/mL, hs-CRP and ASCVD score. Statistical analyses were performed using SPSS version 15.0 (SPSS Inc., Chicago, Illinois).

**Table 1:** Baseline characteristics of patients with and without coronary artery disease

Characteristics	NCAD (N = 13)	CAD (N = 35)	*p*
Male sex n (%)	4 (30.7)	17 (48.5)	0.298
Age (years)	59.6 ± 6.0	56.9 ± 7.5	0.240
BMI	24.52 ± 4.93	27.92 ± 4.64	0.037
BMI >25 (%)	4 (30.7)	28 (82.3)	0.001
Smoking (%)	6 (46.1)	8 (22.8)	0.136
Abdominal circumference (cm)	88.23 ± 11.78	95.59 ± 8.71	0.059
Presence of hypertension (%)	7 (53.8)	9 (25.7)	0.254
Metabolic syndrome n (%)	3 (23.0)	15 (44.1)	0.186
ASCVD risk score (mean ± SD)	10.58 ± 7.62	9.72 ± 6.54	0.706
Previous AMI n (%)	1 (7.69)	5 (14.2)	0.547
Systolic blood pressure (mmHg)	134.94 ± 16.60	138.87 ± 22.52	0.571
Diastolic blood pressure (mmHg)	73.08 ± 16.53	80.80 ± 11.69	0.084
Serum creatinine (mg/dL)	0.68 ± 0.12	0.81 ± 0.22	0.057
UAlb/Ucre (mg/g)	13.5 ± 24.5	24.9 ± 39.8	0.165
Fasting plasma glucose (mg/dL)	93.31 ± 10.59	92.82 ± 8.80	0.878
HbA_1_c (%)	5.60 ± 0.37	5.68 ± 0.33	0.493
Fasting insulin (µU/mL)	9.21 ± 4.20	12.04 ± 6.64	0.139
HOMA-IR	2.18 ± 1.16	2.84 ± 1.65	0.250
Total cholesterol (mg/dL)	173.21 ± 63.13	179.97 ± 39.19	0.664
HDLc (mg/dL)	49.62 ± 11.4	43.39 ± 10.6	0.097
Triglycerides (mg/dL)	107.15 ± 56.64	132.79 ± 82.84	0.318
hs-CRP (mg/L)	4.59 ± 5.74	5.74 ± 6.01	0.565
Alanina transferase (ALT) (mg/dL)	19.15 ± 3.80	24.82 ± 26.54	0.455
On ASA n (%)	6 (46.1)	22 (70.9)	0.198
On statin n (%)	7 (53.8)	24 (77.4)	0.096

*NCAD* patients without coronary artery disease. *CAD* patients with coronary artery disease. UAlb/Ucre was the ratio between urinary albumin concentration and urinary creatinine concentrations. Data are expressed as mean ± SD for all continuous and parametric variables. UAlb/Ucre (mg/g) data were expressed as median and 95% confidence interval

*BMI* body mass index (Kg/cm^2^), *ASCVD* atherosclerotic cardiovascular disease, *AMI* acute myocardial infarction, *HbA1c* glycated hemoglobin, *HOMA-IR* homeostasis model assessment resistance, *HDL*_*c*_ high density lipoprotein cholesterol (mg/dL), *GPT* alanine aminotransferase, *ASA* acetyl salicilic acid, *Ins* insulin, *HbA1c* glycated haemoglobin, *hs-CRP* high-sensitive C-reactive protein (mg/L)

## Results

After exclusion criteria and missing at random losses, 48 patients were included in the study. The prevalence of CAD was 72.9%, with a mean age of the whole group was 57.1 (±7.1) years old. Groups CAD and NCAD were similar in respect of age, gender, smoking status, hypertension, metabolic syndrome, renal function, lipid profile, HbA_1_c, HOMA-IR and statin use. CAD group had a higher prevalence of overweight and obesity (p = 0.001), a greater mean BMI (p = 0.037) and abdominal circumference (p = 0.059). Clinical characteristics of patients are shown in Table 1.

**Fig. 1:**
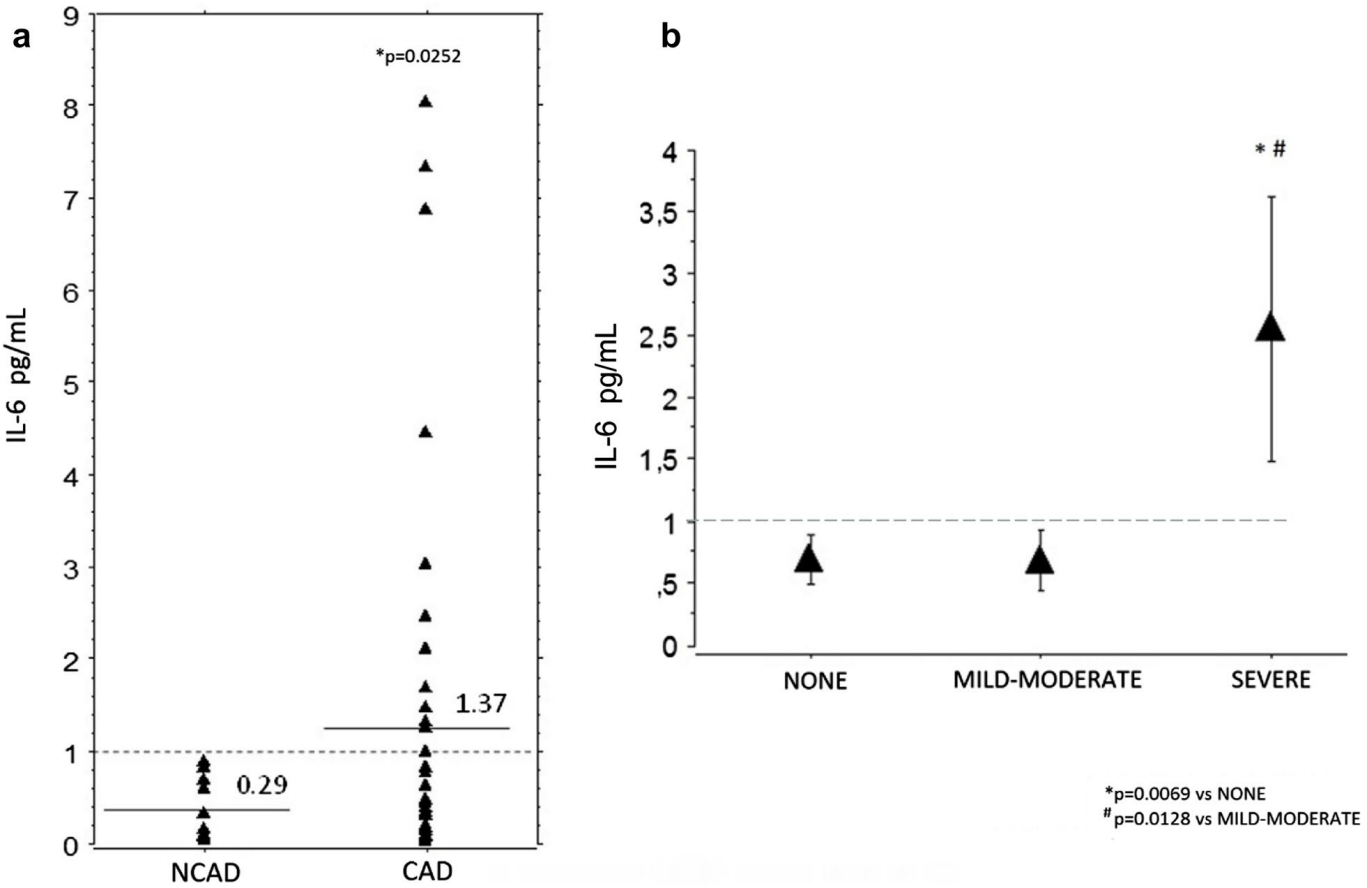
**a** Distribution interleukin-6 (IL-6) levels (pg/mL) in patients with (CAD) or without (NCAD). Significant coronary artery disease was defined as at least one vessel with more than 30% of stenosis at coronary angiography. *Solid lines* indicate the mean. **b** IL-6 levels (pg/mL) in patients according to the score of severity for coronary artery disease. Patients were divided into: NONE (score zero); MILD TO MODERATE (score 1-15); SEVERE (score 6-17). Data are expressed as mean ± standard deviation. *Dashed lines* indicate the cut-off value used in the study

The presence of CAD was associated with higher mean levels of IL-6 (1.37 vs 0.29, p = 0.0252) (Fig. 1a), IL-6 >1.0 pg/mL. was present in 12 (25%) of patients. According to anatomic CAD burden score, from a total of 48 patients included, 24 (50%) had zero score (NONE), 15 (31%) patients had score 1–5 (MILD–MODERATE), and 9 (19%) patients had score 6–17 (SEVERE) CAD. The SEVERE group had progressively increased IL-6 levels compared to both MILD–MODERATE (p = 0.013) and NONE groups (p = 0.007) (Fig. 1b).

Among 12 patients with high IL-6 levels (IL-6 >1.0 pg/mL), all (100%) had CAD while 23 (63.8%) among 36 patients with IL-6 <1.0 pg/mL had CAD. (Chi-squared 5.943, p = 0.0148). The positive predictive value (PPV) in patients with IL-6 >1.0 pg/mL was 100%. The mean ASCVD risk score in 10 years of the entire group was 10.0 ± 6.8%, without differences between groups. According to ASCVD score, among the 48 patients included, 21 (43.7%) were at a low-risk, 24 (50%) were at intermediate risk and 3 (6.2%) were at high-risk. Of the 45 patients at low-intermediate ASCVD risk score, 12 (26.6%) with IL-6 >1.0 pg/mL were re-classified into a high-risk category.

**Fig. 2:**
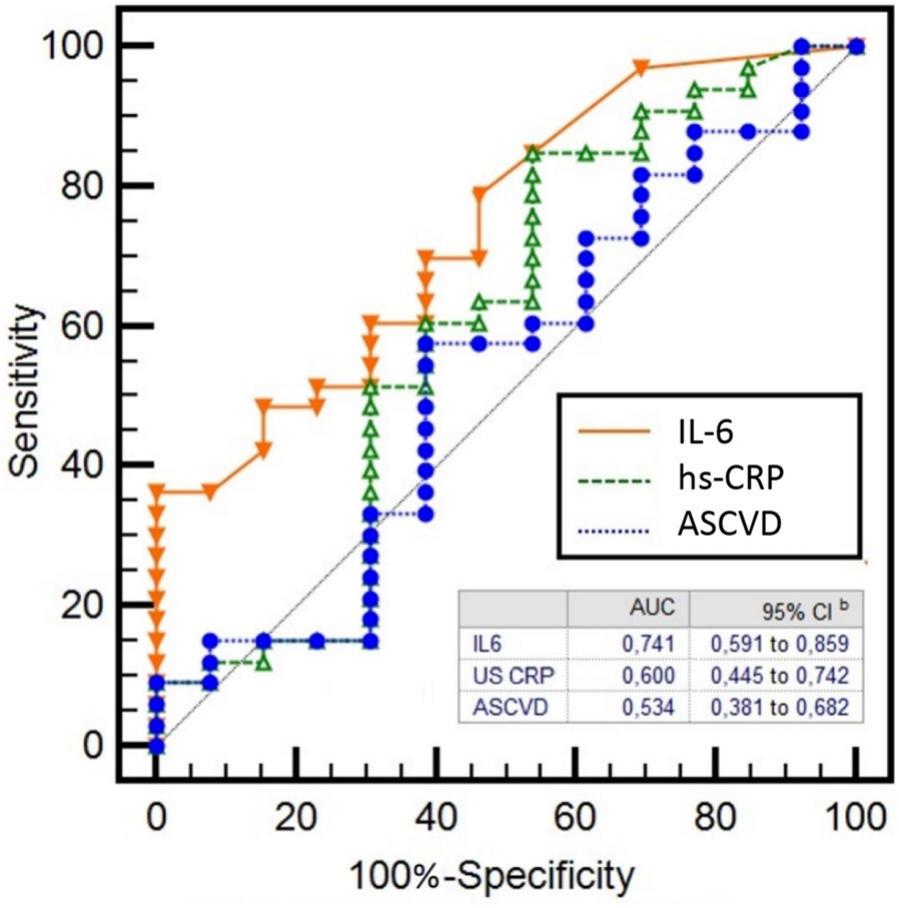
Comparison between ROC curves of interleukin-6 (IL-6), ASCVD score and high-sensitive C-reactive protein (hs-CRP). Data are expresssed as *area under the curve* (AUC) and 95% confidence interval

The area under the receiver operating characteristic curve (AUC), compared the performance of IL-6 and ASCVD risk score and indicates increased accuracy with IL-6. Mean AUC for IL-6, ASCVD and hs-CRP were respectively: 0.74 (95% CI: 0.57–0.84), 0.54 (95% CI: 0.38–0.68) and 0.60 (95% CI: 0.44–0.74). This represents an increase of 38% in accuracy with IL-6 compared to ASCVD, while only 12% when hs-CRP is compared to ASCVD (Fig. 2).

## Discussion

The present study indicates that in intermediate risk patients referred for coronary angiography, a serum IL-6 level above 1 pg/mL is highly predictive for CAD. IL-6 is also an indicator of the severity of atherosclerotic disease. This preliminary study is, to our knowledge, the first to evaluate the association of increased levels of serum IL-6 to the burden of atherosclerotic disease in intermediate-risk population.

Usually, hs-CRP levels are used to re-classify patients at intermediate ASCVD risk. In this scenario, a single determination of IL-6 above 1 pg/mL increased in 38% the accuracy of ASCVD score alone for detecting significant CAD, changing 30% of intermediate risk according to ASCVD score, to a higher risk condition due to the presence of CAD at angiography. In this regard, in the present study, IL-6 had a better performance when compared to hs-CRP, as seen by the increment of the area under the curve in the ROC curve.

Previous studies have observed the association between IL-6 gene polymorphisms and coronary artery disease. A recent meta-analysis [17] and other studies found associations between IL-6 levels and CAD severity, coronary events, mortality and progression to heart failure [12, 13, 18, 19]. Shirai et al. [20] have shown that patients with pre-existing CAD had higher levels of IL-6 compared to patients without CAD, which were not affected by the pharmacotherapy used for treating CAD. None of these studies, however, found a direct association between IL-6 levels and CAD extension.

Inflammation plays a pivotal role in atherosclerosis. Several inflammatory biomarkers have been extensively studied and have found to predict the development of CAD [14, 21, 22]. Remarkably, hs-CRP is the most important related inflammatory biomarker with an increased risk of CAD development. However, IL-6, seems to be a more likely causal factor of CAD if compared to hs-CRP [23, 24]. In our analysis, the area under the ROC curve of IL-6 was larger than hs-CRP to predict CAD, indicating that it may be more accurate. This may be explained due to the fact that IL-6 plays an earlier and more central role in pro-inflammatory regulation process.

Among several mechanisms by which statins reduce cardiovascular outcomes, reducing inflammatory response is probably one of the most accepted. Oka et al. [25] have found that patients who received atorvastatin for 12 weeks had lower IL-6 levels compared to control group. Another study in patients with rheumatoid arthritis found that tocilizumab (a monoclonal antibody that competitively inhibits IL-6 receptor) improved endothelial dysfunction in patients receiving this drug [26]. These are all indirect evidence suggesting that IL-6 plays a role in the development of CAD.

Another important finding in our study was the strong positive predictive value of high IL-6 levels to detect the presence of CAD in patients referred to coronary angiography. Our data suggest that a high IL-6 serum level in non-diabetic overweight patients, at intermediate ASCVD risk can be useful to indicate a higher risk condition and the need for a more invasive approach. Lindmark et al. [27] have tested this hypothesis in patients with unstable coronary artery disease and found that high circulating IL-6 identifies patients who benefit most from a strategy of early invasive management. Differently than IL-6, hs-CRP has a strong negative predictive value in patients with chest pain in the emergency room [28]. Testing the combination of these two inflammatory markers could bring interesting results.

An important strength of the present study was the highly homogeneous intermediate-risk population obtained from an otherwise presumed high-risk population, which resulted in a high powered study (β=0.93, α < 0.05, two-tailed). This allowed conclusions regarding the impact of high IL-6 levels in a lower risk population usually presenting to coronary angiography. Although we have not studied the occurrence of clinical cardiovascular outcomes, there is evidence demonstrating that minor degrees of coronary stenosis (such as 20%) may be predictive of long-term mortality, when compared to the absence of any epicardial coronary stenosis [29].

The main limitations of the present study are thesmall sample of 48 patients and its cross-sectional design, which does not allow cause-and-effect relationship inferences, as it may suffer impact from covariates not measured in the study. We believe, however, that the high power obtained with a small sample may be an indication that IL-6 may become an important predictor for CAD in future larger longitudinal studies.

In conclusion, an elevated IL-6 above 1 pg/mL in intermediate cardiovascular risk population submitted to coronary angiography may be highly predictive of CAD, being associated with the burden of atherosclerosis. IL-6 may be a useful biomarker for detecting significant CAD and to reclassify patients at intermediate ASCVD risk score into a higher risk category. Further long-term clinical trial with IL-6 studies are than warranted to confirm these findings.
